# CT-guided high-dose-rate brachytherapy ablation of HCC patients with portal vein tumor thrombosis

**DOI:** 10.1186/s41747-025-00564-3

**Published:** 2025-02-14

**Authors:** Timo Alexander Auer, Marie-Luise Helene Hildegard Ranner-Hafferl, Melina Anhamm, Georg Böning, Uli Fehrenbach, Raphael Mohr, Dominik Geisel, Roman Kloeckner, Bernhard Gebauer, Federico Collettini

**Affiliations:** 1 Department of Radiology, Charité—Universitätsmedizin Berlin, Augustenburger Platz 1, 13353 Berlin, Germany; 2 Berlin Institute of Health (BIH), Anna-Louisa-Karsch 2, 10178 Berlin, Germany; 3 Department of Hepatology and Gastroenterology, Charité—Universitätsmedizin Berlin, Augustenburger Platz 1, 13353 Berlin, Germany; 4 Institute of Interventional Radiology, University Hospital Schleswig-Holstein Campus Lübeck, Lübeck, Germany

**Keywords:** Brachytherapy, Carcinoma (hepatocellular), Portal vein, Radiology (interventional), Thrombosis

## Abstract

**Background:**

We assessed the safety and efficacy of computed tomography (CT)-guided high-dose-rate (HDR) brachytherapy in treating hepatocellular carcinoma (HCC) with portal vein tumor thrombosis (PVTT).

**Methods:**

From January 2010 to January 2022, 56 patients (median age 67.5 years) with HCC and PVTT underwent 64 procedures. PVTT was further classified according to the Japan liver cancer study group into VP1–VP4. Tumor response was evaluated by cross-sectional imaging 6 weeks after CT-guided HDR brachytherapy and every 3 months thereafter. Local tumor control (LTC), progression-free survival (PFS), and overall survival (OS) were assessed using Kaplan–Meier curves. The severity of procedure-related complications was classified according to the Society of Interventional Radiology guidelines.

**Results:**

Patients were available for imaging evaluation for a median follow-up of 14.0 months. The median diameter of the largest lesion was 56 mm. Estimated median PFS, LTC, and OS were 7.0 (95% CI 5.0–13.0), 14.0 (95% CI 7.0–21.0), and 20.0 (95% CI 13.0–26.0) months respectively. Actuarial 1-, 2-, and 3-year OS rates were 66%, 41%, and 27%, respectively. Subclassified for VP1, VP2, VP3, and VP4 estimated OS was 38.0 (95% CI 9.0-Not-a-number), 21.5 (95% CI 15.0–25.0), 15.0 (95% CI 7.0–33.0), and 13.0 (95% CI 6.0–34.0) months, respectively. Considering the 64 procedures, we recorded no complications for 49 (76.6%), mild-to-moderate complications for 12 (18.8%), and major complications for 3 (4.7%).

**Conclusion:**

CT-guided HDR brachytherapy was safe and effective for locoregional treatment in patients with advanced HCC due to PVTT, achieving long-lasting local tumor control.

**Relevance statement:**

CT-guided HDR brachytherapy is an option to be considered for locoregional treatment of patients with advanced HCC due to PVTT.

**Key Points:**

- Evaluation of CT-guided high-dose-rate (HDR) brachytherapy in treating HCC patients with portal vein tumor thrombosis (PVTT).
- Median OS was 20.0 months ranging between 13.0 and 38.0 months.
- CT-guided HDR brachytherapy seems to be a safe and effective treatment option in HCC patients with PVTT.

**Graphical Abstract:**

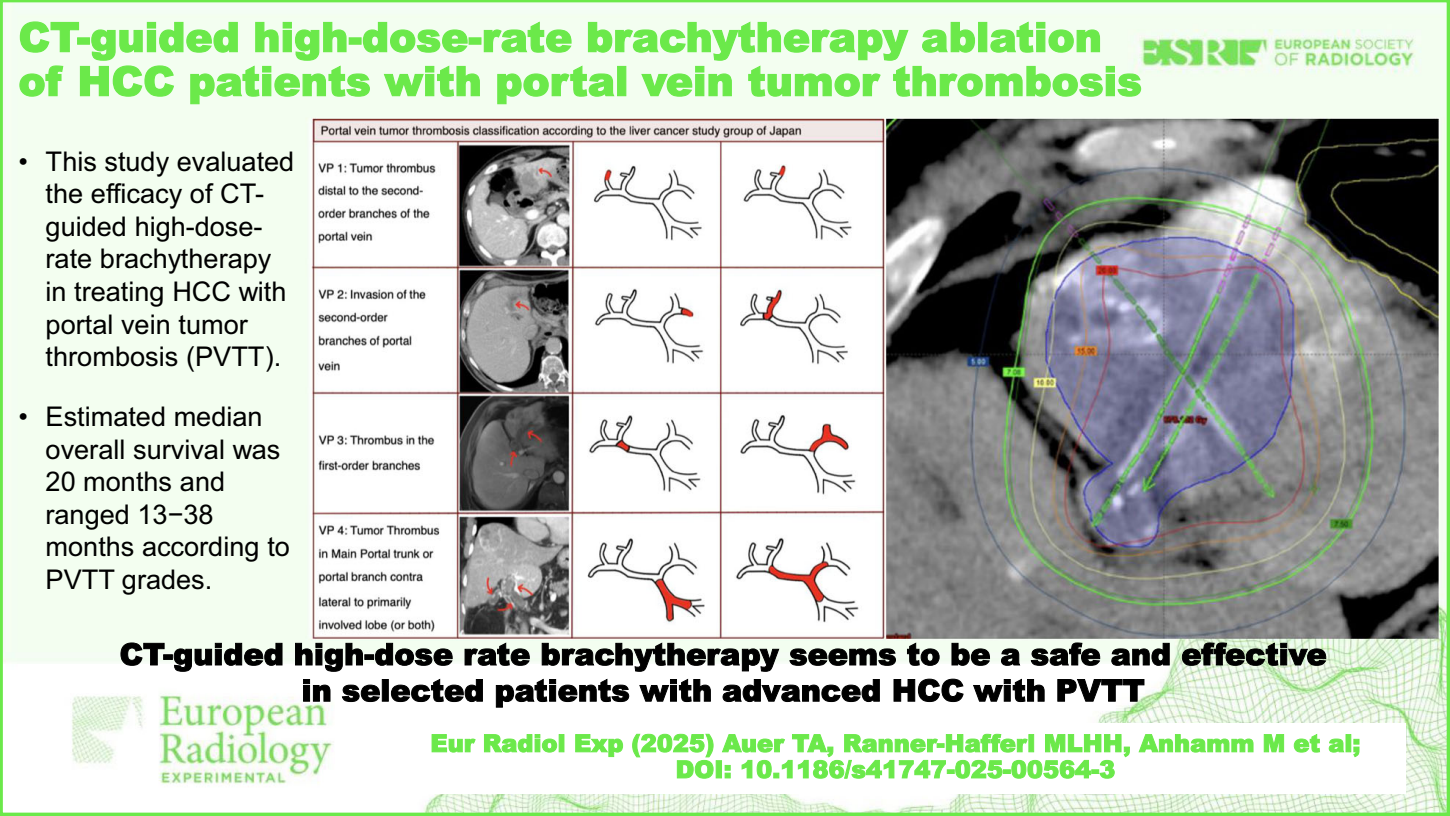

## Background

Hepatocellular carcinoma (HCC) is the sixth most common cancer and the third most common cause of cancer-related death worldwide [1]. It is known for its pronounced ability to invade and grow within the hepatic vasculature. Consequently, around 30% of patients display macrovascular invasion at the time of diagnosis [2]. Portal vein tumor thrombosis (PVTT) is the most common form of HCC macrovascular invasion [3].

According to the Barcelona Clinic Liver Cancer (BCLC) system, patients with PVTT are classified as having “advanced” HCC (BCLC-C), and the only recommended antitumoral treatment is systemic therapy with a combination of atezolizumab and bevacizumab or durvalumab and tremelimumab [4, 5]. However, reported survival rates in patients with BCLC C disease due to PVTT treated with systemic treatments are particularly low (around only 14 months) [6].

The limited survival benefit provided by current systemic treatments in patients with macrovascular invasion highlights the importance of investigating alternative, more nuanced therapeutic approaches that consider the extent of PVTT. While systemic treatment seems to be the most appropriate therapy for patients with more extensive PVTT, more aggressive liver-directed treatments have shown to be safe and effective particularly in patients with segmental PVTT [7]. In recent years, modern radiation-based treatments have gained significant interest in the management of HCC patients with PVTT. Hypofractionated image-guided radiation therapy, such as stereotactic body radiotherapy (SBRT), has been shown to be well tolerated, with acceptable toxicities and promising local control rates [8].

CT-guided high-dose-rate (HDR) brachytherapy is a radioablative technique in which an ^192^Ir source is temporarily inserted into the tumor through brachytherapy applicators placed percutaneously under image guidance [9]. In prior clinical studies, HDR brachytherapy has demonstrated high rates of local tumor control (LTC), with no strict restrictions regarding tumor location or size [10, 11]. Moreover, being a non-thermal ablation technique, HDR brachytherapy is not affected by the heat-sink effect caused by vascular proximity and offers the potential to treat tumors located near temperature-sensitive tissues, such as large bile ducts or gastrointestinal structures [10–12]. In a recent study, CT-guided HDR brachytherapy has been shown to achieve improved survival outcomes compared with transarterial chemoembolization (TACE) in treatment naïve patients with unresectable HCC [13].

The purpose of the present study was to assess the safety and efficacy of CT-guided HDR brachytherapy in treating HCC patients with PVTT.

## Methods

### Study period, inclusion/exclusion criteria, and ethics

In the present study we retrospectively collected and analyzed the data of all consecutive HCC patients with PVTT treated with CT-guided HDR brachytherapy at our institution between January 2010 and January 2022. All patients were discussed in the dedicated institutional interdisciplinary hepatobiliary tumor board, which considered potential alternative therapies and established the indication for CT-guided HDR brachytherapy.

Our institutional inclusion criteria for this treatment option comprises: (1) preserved liver function (Child-Pugh class A or B); (2) total serum bilirubin < 2 mg/dL; (3) platelet count > 50,000/μL; (4) prothrombin time > 50%; and (5) partial thromboplastin time < 50 s. If needed, the hemostatic function was corrected (*e.g*., with platelet concentrates administration), and ascites were drained before treatment. Exclusion criteria include: (1) absence of limited or progressive extrahepatic disease; patients with limited extrahepatic disease confined to a single organ system, with up to three lymph nodes (≤ 2 cm short-axis diameter each) or a solitary non-lymphoid metastasis (≤ 2 cm short-axis diameter) were included [14]; (2) multifocal or diffuse intrahepatic disease (more than five lesions); in patients with up to five lesions, no upper limit was placed upon (largest) tumor diameter.

**Fig. 1:**
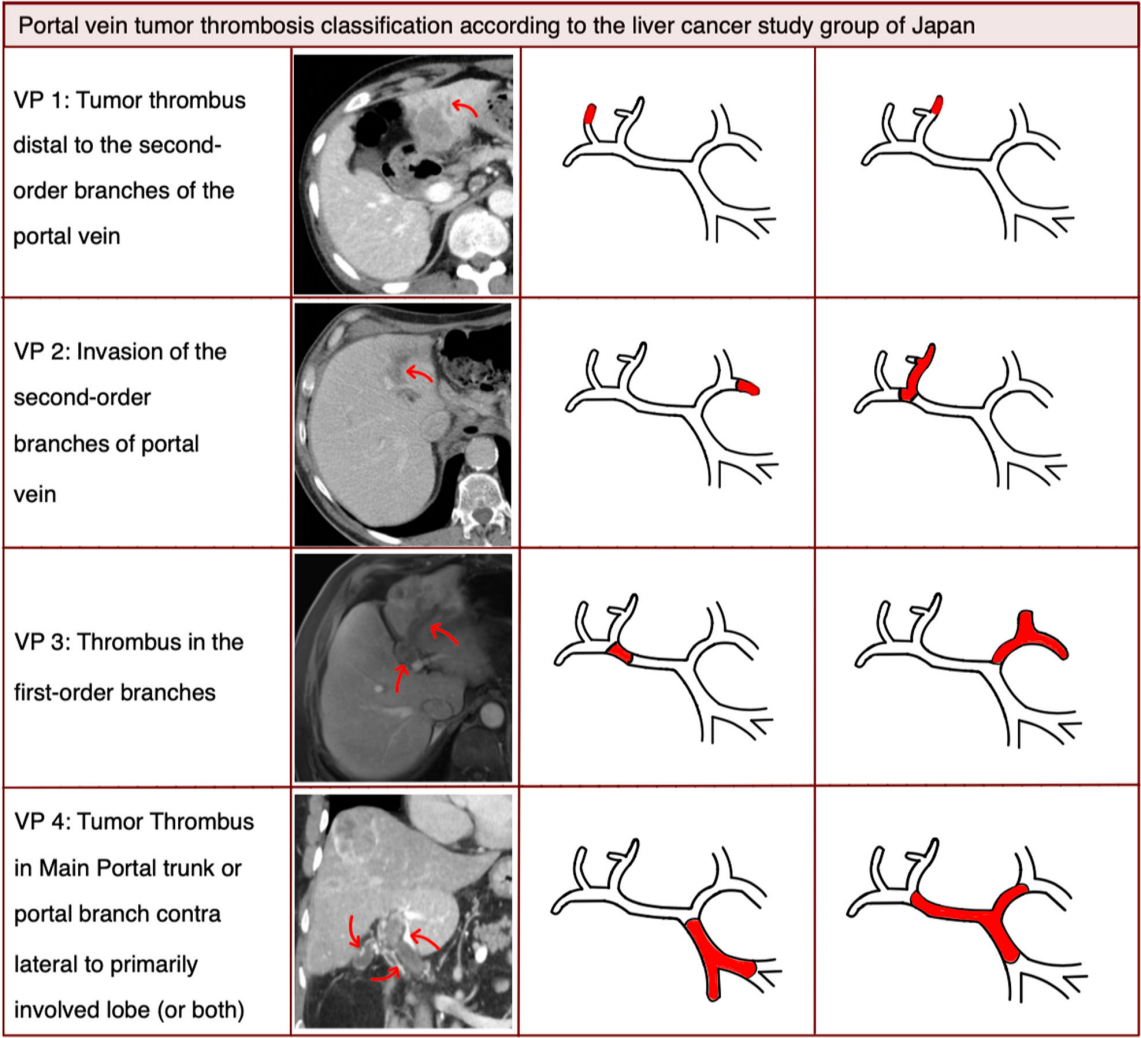
Portal venous tumor thrombosis classification according to the Japan Liver Cancer Study group (own schematic drawing)

Prior to treatment, each patient was given detailed information on the procedure and possible complications. Patients were classified according to the PVTT classification of the Japan liver cancer study group (Fig. 1). Approval for this study was granted by the local ethics committee (EA1/163/22). The retrospective design of the study led to a waiver of the necessity for informed consent.

### Interventional technique

The interventional technique has been described in detail elsewhere [13]. Briefly, percutaneous catheter placement was achieved using CT fluoroscopy guidance (Somatom Definition AS, Siemens, Erlangen, Germany) under analgosedation (midazolam & fentanyl) and local anesthesia at the puncture site (lidocaine). A 17-G trocar puncture needle was advanced into the tumor, exchanged over a stiff guide wire (Amplatz, Boston Scientific, Malborough, USA) for a flexible 6-F catheter sheath (Avanti+, Cordis, Hialeah, Florida, USA) using Seldinger’s technique. The angiographic guide wire was then removed, and a closed-end 6-F afterloading catheter (Primed™, Halberstadt Medizintechnik GmbH, Halberstadt, Germany) was introduced into the catheter sheath.

After securing the catheter(s) with skin sutures, a contrast-enhanced CT scan of the liver was conducted to verify the correct catheter positioning within the tumor and for treatment planning. Computer-based three-dimensional treatment planning was carried out on a dedicated workstation using the acquired dataset and the afterloading planning software Brachyvision™ (Gammamed™, Varian, Palo Alto, CA, USA). All afterloading catheters were digitized from the tip to the body exit point.

Subsequently, the clinical target volume (CTV) and all risk structures (*e.g*., stomach, bowel, spinal cord, hepatic hilum) were delineated. The CTV was defined by the visible tumor borders in contrast-enhanced CT scans, including the enhancing rim. Striving for complete enclosure of the target volume while concurrently preserving the risk structures, a semiautomatic procedure was utilized to calculate and optimize source dwell points and times for the ^192^Ir source inside the afterloading catheters. All irradiations were conducted as single-fraction irradiations in the afterloading technique using a ^192^Ir radiation source with a nominal activity of 10 Ci. The minimum dose to cover the CTV was 20 Gy.

To prevent radiation-induced liver disease, irradiation was planned so that the irradiated liver volume with a dose ≥ 10 Gy/mm^3^ did not exceed one-third of the total liver volume. If exposure of the gastric wall or duodenal mucosa surpassed the critical dose of 10 Gy/mm^3^ of the risk organ, proton pump inhibitors (pantoprazole 40 mg) were prescribed for 6 weeks. The limiting doses for the respective organs are 12 Gy dose in 1 mL and 15 Gy in 0.1 mL for the small intestine, colon, and stomach, 15 Gy in 1 mL and 18 Gy in 0.1 mL for the eosophagus, 10 Gy in 1 mL and 12 Gy in 0.1 mL for the spinal cord, 18 Gy in 1 mL and 20 Gy in 0.1 mL for hilar structure and 10 Gy for the body skin surface. In the case of the liver, the volume irradiated with 10 Gy is crucial, especially in relation to the total liver volume (a maximum of one-third of the liver volume should receive ≥ 10 Gy). Following irradiation, the brachytherapy catheters were carefully withdrawn, and the puncture channels were sealed with a gelatin sponge torpedo (Gelfoam, Pfizer Inc., New York, NY, USA) to prevent secondary bleeding.

In patients with tumors located immediately below the liver capsule or that infiltrated the capsule and grew exophytically, CT-guided HDR brachytherapy was combined with conventional TACE to reduce the risk of bleeding. Conventional TACE was performed in accordance with the Cardiovascular and Interventional Radiological Society of Europe standards of practice for hepatic transarterial chemoembolization [15]. Briefly, after puncturing the common femoral artery, angiography of the celiac trunk and superior mesenteric artery was conducted to identify any parasitic feeders supplying the HCC lesions. Chemoembolization was then carried out in a superselective manner using 1.7- to 2.5-F microcatheters, administering an anthracycline/oil emulsion including doxorubicin, mitomycin, and lipiodol. As an embolic agent, gelatine sponge slurry was used.

### Follow-up

Treatment response was evaluated using contrast-enhanced MRI at 6 weeks post-treatment and subsequently at 3-month intervals by two experienced radiologists in consensus (T.A.A, 8 years of experience, and F.C., 14 years of experience). Successful ablation and tumor control were assessed on the basis of a multiphase and multisequence 1.5- or 3-T MRI protocol using phased-array body coils.

The standard imaging protocols included unenhanced T2-weighted sequences with and without fat saturation, T1-weighted sequences with and without fat saturation (including in-phase and opposed-phase techniques), and diffusion-weighted imaging with *b*-values of 50 and 800 s/mm^2^. Following intravenous administration of Gd-ethoxibenzyl-diethylenetriamine pentaacetic acid (Primovist or Eovist, Bayer Pharma, Leverkusen, Germany) at a dose of 0.025 mmol/kg body weight, manually injected at a flow rate of 1–2 mL/s, followed by a saline flush), multiphase T1-weighted three-dimensional sequences with fat saturation were acquired during breath-hold, capturing the arterial phase at a fixed delay of 15 s, the portal venous phase with a 50-s delay, and the transitional phase with a delay of 90–120 s. The sequence was repeated in the hepatobiliary phase 20 min after contrast administration.

The Liver Imaging Reporting and Data System−LI-RADS® CT/MRI Radiation Treatment Response Assessment [16] was used to assess the response of the treated lesion in patients who underwent brachytherapy as a stand-alone treatment. Patients who underwent both TACE and brachytherapy were evaluated using the LI-RADS® CT/MRI Nonradiation Treatment Response Assessment [16]. LTC was defined as no mass-like enhancement in the treated lesion or alongside its margin (nonviable) or mass-like enhancements (any degree, any phase), which is stable or decreased in size over time (nonprogressing). Local tumor progression was defined as new or increasing mass-like enhancements (any degree, any phase) plus mild-moderate T2 hyperintensities or diffusion restrictions (any degree). Contrast-enhanced late-phase imaging was used to depict the low signal intensity margin post ablation indicating the loss of hepatocytes ability to uptake liver-specific contrast medium, due to an exposure to an irradiation dose of at least 10 Gy [16, 17]. Progression-free survival (PFS) was determined by the absence of: patient death, new or enlarging intrahepatic lesions, extrahepatic tumor progression, or local progression after CT-guided HDR brachytherapy. Overall survival (OS) was defined as the time from therapy to death or loss of contact. The severity of procedure-related complications was classified following the recommendations of the Society of Interventional Radiology (SIR) [18].

### Statistics

Descriptive statistics were used to present baseline characteristics. All statistical analyses were carried out using XLSTAT (Version 2011.0.01; Addinsoft SARL, New York, NY, USA), using *χ*² and contingency tables to assess proportional distribution. Kaplan–Meier analysis was employed to calculate probabilities for LTC, PFS, and OS.

## Results

### Patients and tumors

Over the 12-year interval, 56 HCC patients with PVTT underwent CT-guided HDR brachytherapy at our institution. Demographic data of the patients are summarized in Table 1. The patient population comprised 76.8% (43/56) of men and 23.2% (13/56) of women ranging in age from 40 to 85 years (median: 67.5 years). All patients had concomitant liver cirrhosis with preserved liver function (Child A: 78.6% (44/56); Child B: 21.4% (12/56)). Cirrhosis was attributable to viral hepatitis (either B or C) in 39.3% (22/56), to alcohol use disorder in 37.5% (21/56), to metabolic dysfunction associated fatty liver disease or steatohepatitis in 17.9% (10/56) and in 7.3% (4/56) of cases, the cause of cirrhosis was classified as cryptogenic. Due to macrovascular invasion, all patients had advanced-stage disease (BCLC stage C). According to the PVTT classification of the liver cancer study group of Japan, 6/56 (10.7%) patients were classified as VP1, 15/56 (28.6%) as VP2, 25/56 (35.7%) patients as VP3, and 15/56 (26.8%) patients as VP4 (Fig. 1) [19]. Extrahepatic disease was present in 6/56 (10.7% %) patients. At the time of CT-guided HDR brachytherapy, thirty-five patients (62.5%) were treatment naïve, and the remaining 21 patients (37.5%) had undergone previous therapies including (1) hepatic resection in 5/21 (24.0%) patients, (2) TACE in 5/21 (24%) patients, (3) ablation 5/21 (24%) patients, (4) transarterial radioembolization (TARE) in 5/21 patients (24%) and (5) systemic treatment (Sorafenib) in one patient (4%). Median tumor diameter was 56 mm (ICR: 35.5–78.5). One to 5 catheters were used per treatment session (median average of 2 (IQR: 1–3) catheters), depending on lesion number, size, shape, and location. The median, minimum tumor-surrounding dose was 20 Gy in 25/56 of patients (45%), 15 Gy in 27/56 of patients (48%), 12 Gy in 3/56 of patients (5%) and 10 Gy in one patient (2%). The mean CTV of the treated tumors was 132.6 mL (range 1.3–829.0 mL). Mean coverage across the different enclosing doses was 93%. In 23 patients (41.1%) CT-guided HDR brachytherapy was combined with cTACE. All treatment related parameters are displayed in Tables 2–4.

**Table 1:** Patient and tumor characteristics

Variables		Number of patients	(%)
Age		Median 67.5 years (range 40–85 years)	
Gender	Male	43	76.8
	Female	13	23.2
Tumor diameter (mm)		Median 56 mm (IQR 35.5–78.5)	
	≥ 35 mm	42	75.0
	< 35 mm	14	25.0
Etiology of HCC	Viral	22	39.3
	ETLC	21	37.5
	NASH	10	17.9
	NOS	4	7.1
Treatment naïve patients	Yes	35	62.5
	No	21	37.5
Child-Pugh score	A	44	78.6
	B	12	21.4
Number of lesions	Multifocal	18	32.1
	≤ 3	38	67.9
Tumor characteristics	Diffuse	12	21.4
	Nodular	44	78.6
Metastasis	Yes	6	10.7
	No	50	89.3
Clinical target volume		Mean 132.6 mL (range 1.3–829 mL)	
Ascites	Yes	16	28.5
	No	40	71.5
PVTT classification	VP1	15	26.8
	VP2	20	35.7
	VP3	15	26.8
	VP4	6	10.7

*HCC* Hepatocellular carcinoma, *ETLC* Ethyl toxic liver cirrhosis, *NASH* Non-alcoholic steatohepatitis, *NOS* Not otherwise specified, *PVTT* Portal vein tumor thrombus, *BCLC* Barcelona Clinic Liver Cancer staging system

**Table 2:** Patient blood values

Variables		Number of patients	(%)
Alpha-1-fetoprotein, ng/mL		Median 42 ng/mL (range 2–520.6 ng/mL)	
	< 10 ng/mL	15	26.8%
	10.1–20 ng/mL	4	7.1%
	20–200 ng/mL	16	28.6%
	≥ 200 ng/mL	16	28.6%
	Unknown	5	8.9%
Albumin, g/L		Median 37.75 g/L (range 26.1–48.4 g/L)	
	≥ 35 g/L	40	71.4%
	< 35 g/L	14	25.0%
	Unknown	2	3.6%
International normalized ratio		Median 1.12 (range 1–1.61)	
	< 1.15	34	60.7%
	≥ 1.15	22	39.3%
Bilirubin, mg/dL		Median 0.84 mg/dL (range 0.21–2.5 mg/dL)	
	< 1.1 mg/dL	32	57.1%
	> 1.1 mg/dL	24	42.9%
Prothrombin ratio, %		Median 83% (range 42–112%)	
	< 100%	49	87.5%
	≥ 100%	5	8.9%
	Unknown	2	3.6%

**Table 3:** Treatment characteristics

Variables		Number of patients	(%)
Therapeutic	CT-HDBRT alone	33	58.9%
modalities	CT-HDBRT & TACE	23	41.1%
Therapeutic modalities prior to CT-HDBRT	Resection	4	7.1%
	TACE	4	7.1%
	TARE	4	7.1%
	Ablation	4	7.1%
	Systemic treatment	1	1.8%
CT-HDBRT dose, Gy		Median 15 Gy (range 10–20 Gy)	
	≤ 15 Gy	31	55.4%
	> 15 Gy	25	44.6%
CT-HDBRT coverage		Median 99.6% (range 32–100%)	
	< 100%	36	64.3%
	100%	20	35.7%
Number of catheters used in CT-HDBRT	1	16	28.6%
	2	20	35.7%
	3	16	28.6%
	4	3	5.3%
	5	1	1.8%

*CT-HDBRT* CT-guided-high-dose-rate brachytherapy, *TACE* Transarterial chemoembolization

**Table 4:** Previous treatments

Previous treatment	Number (%)
Hepatic resection	5 (24.0)
Transarterial chemoembolization	5 (24.0)
Ablation	5 (24.0)
Transarterial radioembolization	5 (24.0)
Sorafenib	1 (4.0)

### Procedure-related complications

Patients were available for imaging evaluation for a median follow-up of 14.0 months (interquartile range 6.3–25.5). In the total study population (64 procedures in 56 patients), for 49/64 procedures (76.6%) we observed no complications; for 12/64 procedures (18.8%) mild-to-moderate complications (SIR grade 1: *n* = 9; SIR grade 2: *n* = 3); and for 3/64 procedures (4.7%) major complications (SIR grade 3: *n* = 1; SIR grade 4: *n* = 1; SIR grade 5: *n* = 1). Major complications included one infection (grade 3) and 2 bleedings (grade 4 and 5).

### Local tumor control, progression-free survival, and overall survival

**Fig. 2:**
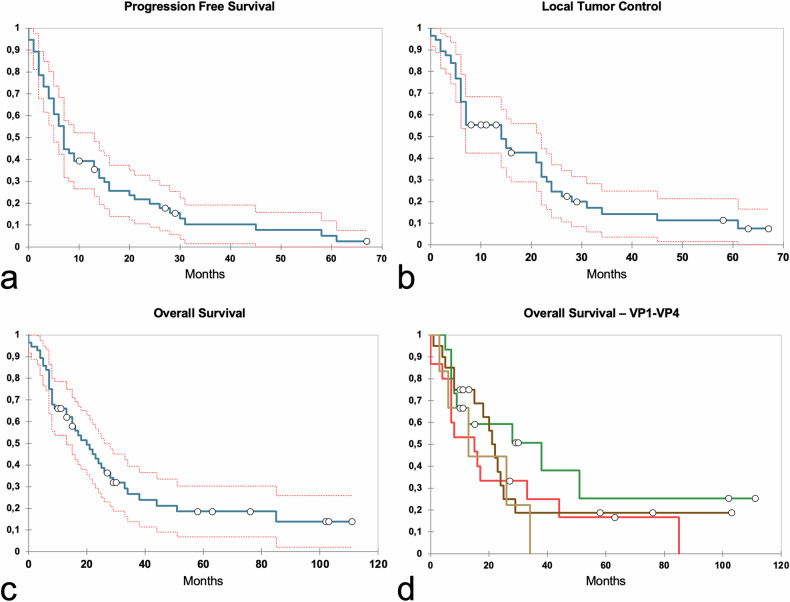
Kaplan–Meier curves for the entire cohort. Red dashed lines indicate 95% confidence intervals: progression-free survival (**a**); local tumor control (**b**). Overall survival is shown in (**c**, **d**), the cohort is stratified according to the portal vein tumor thrombosis subclasses from VP1 to VP4 (Japan Liver Cancer study group: VP1, green; VP2, brown; VP3, red; VP4, beige)

During the follow-up period, 14 out of 56 (25%) tumors treated with CT-guided HDR brachytherapy showed LTC. The estimated median LTC was 14.0 (95% CI 7.0–21.0) months (Fig. 2a). Six of the 14 local progressions were treated by repeated CT-guided HDR brachytherapy and maintained consistent LTC throughout the follow-up period. Of the remaining eight patients with local tumor progression, two were treated with TARE, two with TACE, three patients underwent systemic therapy with sorafenib, Lenvatinib, and the combination of Atezolizumab and Bevacizumab, respectively, and two received the best supportive care.

**Fig. 3:**
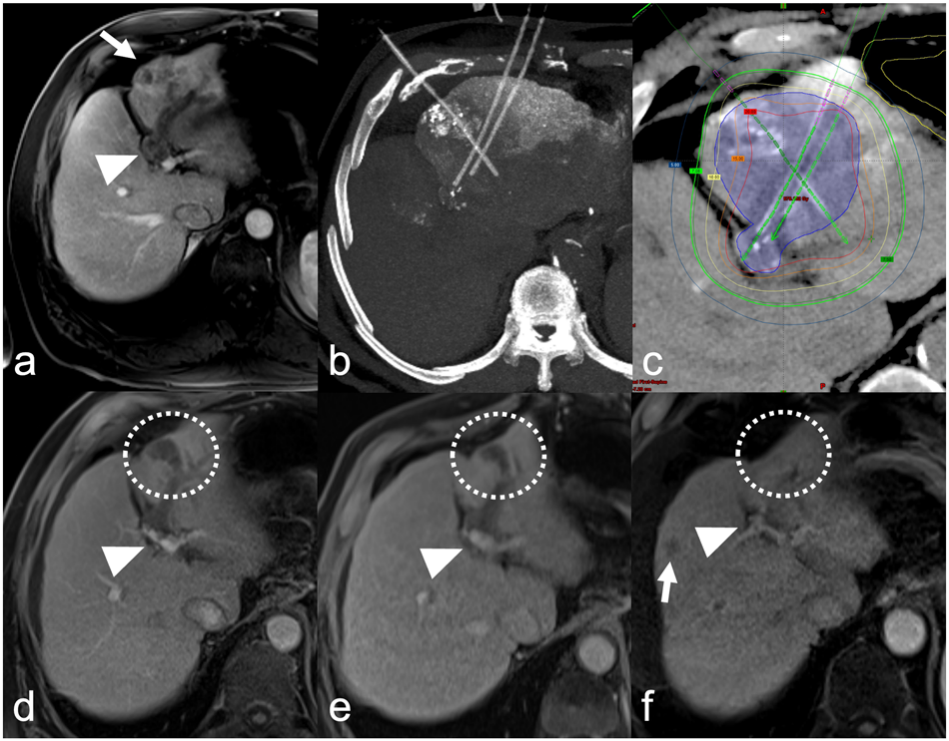
Example images in a 71-year-old male treatment-naive patient with unresectable hepatocellular carcinoma (HCC). **a** Baseline MRI scan (portal venous phase) shows a single 63-mm HCC (arrow) with portal venous tumor thrombus in the left first-order branch (VP3, arrowhead) in the left liver lobe. **b** Maximum intensity projection of a computed tomography scan acquired after placement of three brachytherapy applicators and lipiodol deposition after transarterial chemoembolization the day before. **c** Treatment planning for high-dose-rate irradiation. The region highlighted in blue and bordered by the light blue line represents the 162 mL clinical target volume. The realized minimum dose in the clinical target volume (D100) was 15 Gy. The colored lines around the clinical target volume represent the respective isodose lines: red, 20 Gy; orange, 15 Gy; yellow, 10 Gy; green, 7 Gy; and blue, 5 Gy. Follow-up contrast-enhanced magnetic resonance images at 8 weeks (**d**), 3 weeks (**e**), and 12 months (**f**) show persistent tumor control and progressive tumor shrinkage (dashed circle), while the arrowhead shows portal venous tumor thrombus shrinkage from VP2–3 (**d**), to VP2 (**e**) and VP1 (**f**)

Distant intrahepatic or extrahepatic tumor progression was seen in 23/56 (41%) patients. Estimated median PFS was 7.0 (95% CI 5.0–13.0) months. Actuarial 1-, 2-, and 3-year PFS rates were 36%, 20%, and 10%, respectively (Fig. 2b). Of the 23 patients with distant intrahepatic or extrahepatic tumor progression, eight patients were treated with CT-guided HDR brachytherapy. The remaining patients underwent either systemic therapy (*n* = 8) or BSC (*n* = 7). All patients were included in the survival analysis. Estimated median OS for the entire population was 20.0 (95% CI 13.0–26.0) months. Actuarial 1-, 2-, and 3-year OS rates were 66, 41, and 27%, respectively (Fig. 2c). Survival rates according to the degree of PVTT showed a significant difference between the PVTT-subgroups, with mean survival rates for VP1, VP2, VP3, and VP4 of 38.0 (95% CI 9.0-Not-a-number), 21.5 (95% CI 15.0–25.0), 15.0 (95% CI 7.0–33.0) and 13.0 (95% CI 6.0–34.0) months, respectively (Fig. 2d). Figure 3 presents a representative course in a patient with persistent LTC.

For further subgroup analyses, please also see Table 5 and Table 6.

**Table 5:** Survival rates and subanalyses

Variables		Entire cohort	HD only	HDR and TACE	*p*-value	Treatment naive	Pretreated	*p*-value
LTC, months, median (IQR)		14.0 m (7.0–21.0)	7.0 m 6.0–22.0	16.0 m 7.0–22.0	0.58 8	15.0 m 7.0–24.0	7.0 4.0–16.0	0.23 1
LTP events (%)	Yes No	25.0 (14) 75.0 (42)	85 (28) 15 (5)	74 (17) 16 (6)	−	77 (27) 23 (8)	86 (18) 14 (3)	
PFS, months, median (IQR)		7.0 (5.0–13.0)	7.0 (5.0–13.0)	7.0 (5.0–15.0)	0.815	7.0 (6.0–14.0)	5.0 (4.0–14.0)	0.219
TP events (%)	Yes No	66.1 (37) 33.9 (19)	94 (31) 6 (2)	87 (20) 13 (3)	−	89 (31) 11 (4)	95 (20) 5 (1)	
OS, months, median (IQR) events (%)	Yes No	20.0 (13.0–26.0) 73.2 (41) 26.8 (15)	18.0 (8.0–34.0) 76 (25) 24 (8)	22.0 (13.0–28.0) 70 (16) 30 (7)	0.818	24.0 (13.0–34.0) 71 (25) 29 (10)	17.0 (7.0–23.0) 76 (16) 24 (5)	0.237

*LTC* Local tumor control, *IQR* Interquartile range, *LTP* Local tumor progression, *OS* Overall survival, *PFS* Progression-free survival

**Table 6:** Survival rates divided by portal venous tumor thrombus grade

Variables		V1	V2	V3	V4
LTC, months, median (IQR)		7.0 (6.0–28.0)	15.0 (7.0–22.0)	7.0 (6.0–31.0)	13.0 (6.0–26.0)
LTP events (%)	Yes No	93 (14) 7 (1)	75 (15) 25 (5)	87 (13) 23 (2)	83 (5) 17 (1)
PFS, months, median (IQR)		7.0 (4.0–15.0)	8.5 (5.0–15.0)	5.0 (3.0–13.0)	9.5 (6.0–20.0)
TP events (%)	Yes No	94 (31) 6 (2)	85 (17) 15 (3)	93 (14) 7 (1)	100 (6) 0 (0)
OS, months, median (IQR) events (%)	Yes No	38.0 9.0−NaN 60 (9) 40 (6)	21.5 15.0–25.0 70 (14) 30 (6)	15.0 7.0–33.0 87 (13) 13 (2)	13.0 6.0–34.0 83 (5) 17 (1)

*LTC* Local tumor control, *IQR* Interquartile range, *LTP* Local tumor progression, *NaN* Not-a-number, *OS* Overall survival, *PFS* Progression-free survival

## Discussion

Despite recent improvements in systemic treatments in HCC patients with PVTT, many researchers advocate the use of locoregional treatments in selected patients with PVTT [20]. The results of the present study show that for selected patients with PVTT, CT-guided HDR brachytherapy may be a safe and effective treatment. CT-guided HDR brachytherapy achieved high LTC rates, with 75% of tumors remaining controlled throughout the follow-up period. Tumor control translated in an estimated median OS of 20 months for the entire population and as high as 38 months in patients with VP1 PVTT. Consistent with the literature, more advanced PVTT was associated with poorer survival rates [21].

While HCC is known to be radiosensitive, radiation-based treatments like external beam radiotherapy have not been implemented into therapy regimes for advanced HCC until recent years, primarily due to the liver’s dose tolerance constraints [22, 23]. With the advancements in imaging modalities and radiation therapy techniques, highly accurate, hypofractionated image-guided radiation-based therapies such as intensity-modulated radiation therapy and stereotactic body radiation therapy (SBRT), directed to tumors while avoiding damage to normal liver tissue, have been shown to be well tolerated with acceptable toxicities and promising LCT rates.

In 2013, Bujold et al reported the results of two prospective trials investigating the use of SBRT in 102 HCC patients (55% of whom with PVTT) deemed unsuitable for other locoregional therapies. The median prescription dose was 36 Gy in six fractions. Toxicity ≥ grade 3 was seen in 30% of patients. The median OS of the entire cohort was 17.0 months [24]. In a retrospective comparative study on 104 HCC patients with PVTT Yang et al showed that stereotactic ablative radiotherapy was superior to conventionally fractionated radiotherapy (CFRT) in terms of overall response rate (62.2% *versus* 33.8%, *p*  =  0.003) and in-field PFS rate (69.6% *versus* 32.2%, *p*  =  0.007). The 1-year OS rate (34.9% *versus* 15.3%, *p*  =  0.012) was also significantly higher in the SABR *versus* CFRT group. There was no difference between treatment groups in the incidence of radiation-induced liver disease. SBRT was also tested in combination with other locoregional therapies such as TACE. The largest study in the literature was conducted by Kang et al and involved 101 HCC patients with PVTT who underwent SBRT ± TACE with a median dose of 40.2 Gy. The reported overall tumor response rate was 87.1%; the median survival time was up to 17 months. Compared to the survival rates we reported, our results are similar or superior; however, it is important to consider that these patient groups are heterogeneous.

Another locoregional therapy that has been evaluated in HCC patients with PVTT is TARE, a form of brachytherapy in which small, intra-arterially injected microspheres loaded with a radioisotope of ^90^Y of ^166^Ho serve as a source for internal radiation. Compared to TACE, TARE poses substantially less risk of ischemia to the liver parenchyma in the presence of portal vein thrombosis, making it a more attractive treatment option for this subgroup of patients with unresectable HCC. Abouchaleh et al evaluated the use of TARE in 185 HCC patients with portal vein thrombosis. Depending on Child-Pugh stage and location of portal vein thrombosis, medial survival ranged from 14.3 months in patients with Child-Pugh A and segmental thrombosis to 3.4 months in patients with Child-Pugh B8 and main trunk thrombosis [25].

A significant improvement in outcomes for HCC patients with PVTT treated with TARE has been achieved through the introduction of personalized dosimetry. This approach delivers higher radiation doses to the tumor than standard dosimetry (> 205 Gy *versus* 120 Gy) without significantly increasing liver adverse events. Recently, the phase II DOSISPHERE-01 trial showed that HCC patients with PVTT treated with personalized dosimetry had better OS compared to those treated with standard TARE (22.9 *versus* 9.5 months), with up to 12.2% of patients being downstaged to resection [26]. The DOSISPHERE-01 trial clearly highlights the importance of delivering high doses of radiation to the tumor while minimizing to the surrounding liver parenchyma [26]. This is precisely the objective of the CT-guided HDR brachytherapy technique presented here.

To the best of our knowledge, this is the first study evaluating the use of CT-guided HDR brachytherapy in HCC patients with PVTT. This approach differs from conventional teletherapeutic procedures (SBRT, Cyberknife etc.) in a number of key aspects that turn out to be relevant for the treatment of advanced liver malignancies. During CT-guided HDR brachytherapy, catheters are inserted percutaneously into the tumor enabling delivery of high radiation dosages (> 100 Gy in the tumor centre) in a single fraction with a rapid dose gradient towards the adjacent healthy liver tissue [13, 27]. In a recent study, Walter et al compared CT-guided HDR brachytherapy with SBRT in terms of clinically relevant liver dose exposure and found out that the liver volume exposed to 10 Gy in a single fraction delivered with CT-guided HDR brachytherapy was smaller than the volume exposed to 20 Gy in fractionated SBRT, suggesting a clear advantage of CT-guided HDR brachytherapy for normal liver tissue sparing.

The results of the present study show that HDR brachytherapy is a safe and effective treatment for selected patients with unresectable HCC complicated by PVTT achieving high rates of LTC and estimated median OS rates of up to 38.0 and 21.5 months in patients with segmental (VP1 and VP2) PVTT. Although making indirect comparisons across different studies using various techniques is challenging, the survival outcomes reported here are favorable compared to those reported with SBRT and are comparable to those reported with TARE using personalized dosimetry.

Several limitations need to be acknowledged. First, it is a retrospective, non-comparative analysis of a small group of patients treated at a single institution over an extended period. Second, since the vast majority of patients (78%) had Child-Pugh A cirrhosis, our results are difficult to generalize to the broader population of patients with advanced-stage disease.

In summary, CT-guided HDR brachytherapy was a safe and effective locoregional treatment for achieving long-lasting LTC in selected patients with advanced HCC due to PVTT. The results obtained in the present study indicate that in patients with segmental (VP1 and VP2) PVTT, HDR brachytherapy can achieve survival rates similar to those obtained with other locoregional therapies in patients with BCLC B disease. To our knowledge, this is the first study to investigate CT-guided HDR brachytherapy in advanced-stage HCC. Larger, prospective studies are needed to confirm these early, promising results for this challenging subset of patients.

## Data Availability

Accessible on reasonable request.

## Abbreviations

BCLC: Barcelona Clinic Liver Cancer (BCLC)
CFRT: Conventionally fractionated radiotherapy
CTV: Clinical target volume
HCC: Hepatocellular carcinoma
HDR: High-dose-rate
LTC: Local tumor control
MRI: Magnetic resonance imaging
OS: Overall survival
PFS: Progression-free survival
PVTT: Portal vein tumor thrombosis
SBRT: Stereotactic body radiotherapy
SIR: Society of Interventional Radiology
TACE: Transarterial chemoembolization
TARE: Transarterial radioembolization
